# Genome-Wide Identification and Analysis of Maize *DnaJ* Family Genes in Response to Salt, Heat, and Cold at the Seedling Stage

**DOI:** 10.3390/plants13172488

**Published:** 2024-09-05

**Authors:** Gang Li, Ziqiang Chen, Xinrui Guo, Dagang Tian, Chenchen Li, Min Lin, Changquan Hu, Jingwan Yan

**Affiliations:** Biotechnology Research Institute, Fujian Academy of Agricultural Sciences, Fuzhou 350003, China; ligang4439@163.com (G.L.); czq@fjage.org (Z.C.); guoxinrui1@163.com (X.G.); tdg@fjage.org (D.T.); lcc823j@163.com (C.L.); linmin_0916@163.com (M.L.)

**Keywords:** *DnaJ* gene family, abiotic stress, maize, gene expression

## Abstract

DnaJ proteins, also known as HSP40s, play a key role in plant growth and development, and response to environmental stress. However, little comprehensive research has been conducted on the *DnaJ* gene family in maize. Here, we identify 91 *ZmDnaJ* genes from maize, which are likely distributed in the chloroplast, nucleus, and cytoplasm. Our analysis revealed that *ZmDnaJs* were classified into three types, with conserved protein motifs and gene structures within the same type, particularly among members of the same subfamily. Gene duplication events have likely contributed to the expansion of the *ZmDnaJ* family in maize. Analysis of cis-regulatory elements in *ZmDnaJ* promoters suggested involvement in stress responses, growth and development, and phytohormone sensitivity in maize. Specifically, four cis-acting regulatory elements associated with stress responses and phytohormone regulation indicated a role in adaptation. RNA-seq analysis showed constitutive expression of most *ZmDnaJ* genes, some specifically in pollen and endosperm. More importantly, certain genes also responded to salt, heat, and cold stresses, indicating potential interaction between stress regulatory networks. Furthermore, early responses to heat stress varied among five inbred lines, with upregulation of almost tested *ZmDnaJ* genes in B73 and B104 after 6 h, and fewer genes upregulated in QB1314, MD108, and Zheng58. After 72 h, most *ZmDnaJ* genes in the heat-sensitive inbred lines (B73 and B104) returned to normal levels, while many genes, including ZmDnaJ55, 79, 88, 90, and 91, remained upregulated in the heat-tolerant inbred lines (QB1314, MD108, and Zheng58) suggesting a synergistic function for prolonged protection against heat stress. In conclusion, our study provides a comprehensive analysis of the *ZmDnaJ* family in maize and demonstrates a correlation between heat stress tolerance and the regulation of gene expression within this family. These offer a theoretical basis for future functional validation of these genes.

## 1. Introduction

Plants are subjected to a range of stresses, including drought, heat, salinity, cold, and pathogens, all of which have the potential to impede their growth and reduce yield [1]. A 1 °C increase in temperature is reported to potentially reduce the production of maize (*Zea mays* L.), wheat (*Triticum aestivum* L.), rice (*Oryza sativa* L.), and soybean (*Glycine max* (L.) *Merr.*) by 7.4%, 6%, 3.2%, and 3.1%, respectively [2]. In response to such stresses, plants produce heat shock proteins (Hsps), which are essential for cell survival during periods of stress [3,4]. Previous studies have shown that Hsps, classified as Hsp100, Hsp90, Hsp70, Hsp60, Hsp40, and Hsp20, based on molecular weight, function in a variety of cellular processes, such as maintaining protein folding, facilitating membrane transport, and disaggregating protein complexes [5,6]. Among these, Hsp40 proteins are the most prevalent in eukaryotic organisms. For example, yeast is reported to have 22 Hsp40 members [7], while *Arabidopsis thaliana* and rice contain 109 and 104 Hsp40 members, respectively [8,9].

Hsp40s, also known as DnaJ proteins (DnaJs), are characterized by a conserved J-domain featuring a histidine/proline/aspartic (HPD) signature motif. This motif is crucial for interacting with Hsp70, which regulates its ATPase activity and facilitates the refolding of stress-damaged proteins [10,11]. Except for the common J-domain, DnaJ proteins exhibit extensive structural diversity. Based on their domains, DnaJ proteins can generally be divided into three types: type I, type II, and type III. Type I DnaJ proteins include three typical domains, namely the J-domain, G/F domain, and C-terminal domain. Type II DnaJ proteins are similar to type I but lack the G/F domain. Type III DnaJ proteins, the most diverse group, present only the J-domain or are accompanied by other diverse domains like the TPR domain, Fe-S cluster, or AT-hook domain, which may lead to more specialized functions [8,9,11]. The different types of DnaJ proteins reflect their functional diversity.

Studies showed that plant DnaJ proteins are widely involved in various biological processes, including chloroplast function and plant stress responses. For example, DJA6 and DJA5 proteins in *Arabidopsis* facilitate chloroplast iron-sulfur cluster biogenesis [12,13]. DnaJ proteins, such as AtJ8, AtJ11, and AtJ20, are essential for optimizing photosynthesis and chloroplast efficiency [14]. In stress responses, the AtJ3 protein in *Arabidopsis* regulates pH homeostasis and influences stress sensitivity, particularly under alkaline conditions and high salt [15]. The tomato SlDnaJ20 protein enhances thermo-tolerance and reduces photosystem II photoinhibition under heat stress [16]. LeCDJ1, a tomato chloroplast-targeted DnaJ protein, could improve tolerance to heat, chilling, and drought stress, as well as resistance to *Pseudomonas solanacearum* [17,18,19]. In addition, two ER-localized DnaJ proteins, ERdj3B and TMS1, are crucial for the development of anthers and pollen tubes under high-temperature stress [20,21,22]. These findings indicate the significance of DnaJ proteins in stress responses.

Currently, identification efforts for the *DnaJ* gene family have primarily focused on *Arabidopsis*, rice, cucumber, and pepper [8,9,23,24]. However, there were no reports on maize, a globally significant crop that frequently faces biotic and abiotic stress. In the present work, we systematically identify and characterize *ZmDnaJs* in the maize genome, including the gene structure, conserved motif, cis-element in the promoter, and phylogenetic relationship. We also analyze the expression patterns of *ZmDnaJs* in various tissues and under abiotic stresses, especially for expression levels under high temperatures. This work provides a thorough examination of the *ZmDnaJ* family in maize and lays the foundation for further investigations to determine whether the *ZmDnaJ* gene family members are involved in the response to environmental stress.

## 2. Results

### 2.1. ZmDnaJ Gene Family in the Maize Genome and Their Physiochemical Properties

In this study, 91 *ZmDnaJ* genes were identified in the maize genome using the hidden Markov model (HMM) of the DnaJ structural domain (PF00226). Based on the Pfam and NCBI CDD databases, we confirmed that these proteins contained the full DnaJ domain. These genes were renamed *ZmDnaJ1*~*ZmDnaJ91* according to their locations on the chromosome (Table S1).

The physicochemical properties of these genes and their corresponding proteins were further analyzed, including gene ID, location, protein length (aa), molecular weight (MW), theoretical isoelectric point (pI), instability index, grand average of hydropathicity, and subcellular location. The number of amino acids ranged greatly from 121aa (ZmDnaJ43) to 2574aa (ZmDnaJ6). The molecular weights ranged from 13,812.54 Da (ZmDnaJ38) to 281,715.97 Da (ZmDnaJ6), pI from 4.41 (ZmDnaJ18) to 10.8 (ZmDnaJ32), and the instability index from 24.94 (ZmDnaJ43) to 97.37 (ZmDnaJ65). The grand average of hydropathicity was <0, indicating that all members of the ZmDnaJ family were hydrophilic proteins. Subcellular localization prediction showed that the majority of ZmDnaJ members were located in the cytoplasm, chloroplast, or nucleus, with few members in the mitochondrion or endoplasmic reticulum or vacuole, indicating their various functions (Table S1).

### 2.2. Phylogenetic and Conserved Domain Analysis of the ZmDnaJ Gene Family

**Figure 1:**
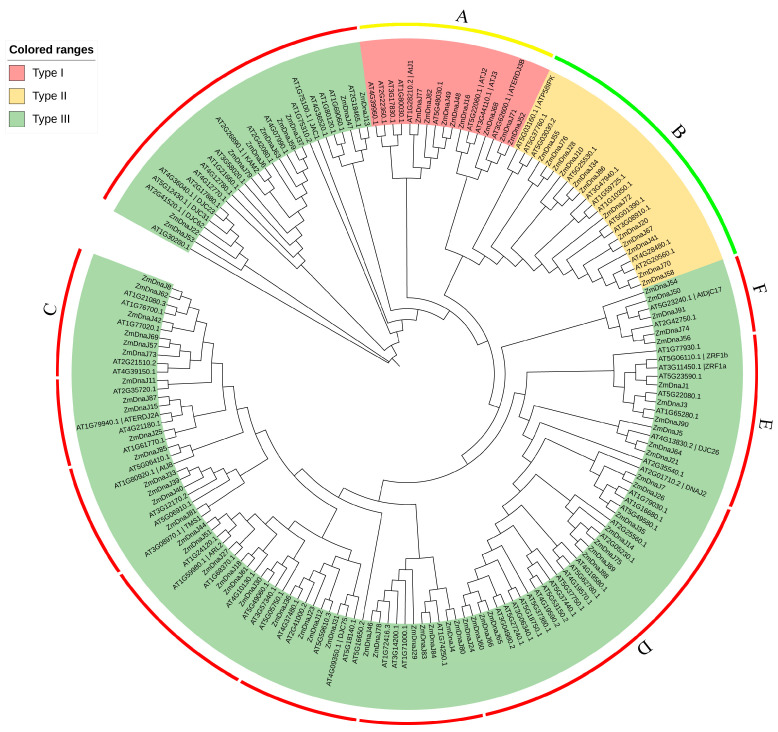
Phylogenetic tree of ZmDnaJ and AtDnaJ proteins. The phylogenetic tree was constructed using MEGA X with 1000 bootstrap replications and an optimal Jones–Taylor–Thornton (JTT) model, incorporating the DnaJ proteins from maize and *Arabidopsis*. The new names for maize proteins are listed in Table S1, and the accession numbers for *Arabidopsis* proteins are based on Rajan et al. [8]. Different background colors represent the three types. A and B represent the DnaJ_C subfamily; C represents the DnaJ-X subfamily; D represents the DUF3444 subfamily; E represents the Jiv90 subfamily; F represents the Fer4_8 subfamily.

To understand the phylogenetic relationships of the *ZmDnaJ* gene family, a phylogenetic tree was constructed based on the 91 ZmDnaJ and 101 *Arabidopsis* DnaJ protein sequences using MEGA-X with the maximum likelihood (ML) method. The phylogenetic tree of *DnaJ* gene members from *Arabidopsis* and maize showed different clades, based on their conserved protein sequences. According to the classification of the *AtDnaJ* members, the maize *ZmDnaJ* members were divided into three types (type I, type II, and type III) with the related *Arabidopsis* homeobox genes. Type I contains nine *AtDnaJs* from *Arabidopsis* and eight *ZmDnaJs* from maize. Type II presents 11 *AtDnaJs* and 12 *ZmDnaJs*. These two types have a close evolutionary relationship, indicating that they are relatively conserved in their evolution and functions. The remaining *DnaJ* genes from *Arabidopsis* and maize were clustered into type III, constituting the largest group. It is worth noting that type III could be further divided into several subgroups, which may exhibit the most diverse functions (Figure 1).

To understand the relationship between evolution and conserved domains among ZmDnaJ proteins, we further analyzed their conserved domains using the NCBI-CDD website. The evolutionary tree of ZmDnaJ proteins was conducted by MEGA-X to distinguish different clades. As shown in Figure S1, we found that ZmDnaJ proteins belonging to types I and II (Figure 1) generally consist of a J-domain followed by a C-terminal domain (DnaJ_C), apart from ZmDnaJ20. In addition to the signature J-domain, type III J-proteins contain diverse domains such as DnaJ-X, DUF3444, TPR domain, Fer4_8 cluster, or Jiv90, which confer additional specificity. These results indicate that the conserved domains of ZmDnaJ proteins within the same group exhibit similar compositions.

### 2.3. Conserved Motif and Gene Structure Analysis of the ZmDnaJ Gene Family

**Figure 2:**
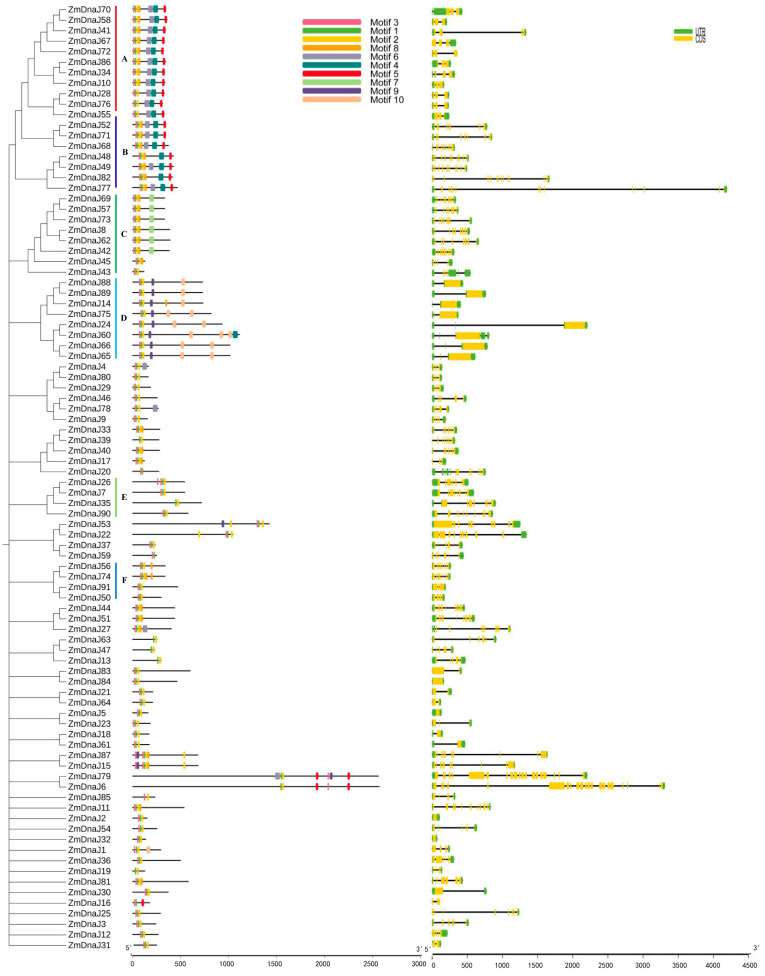
Phylogenetic relationship, conserved motif, and gene structure of *ZmDnaJ* members. Different colored boxes represent various motifs or exons, while black lines indicate non-conserved sequences and introns. A and B represent the DnaJ_C subfamily; C represents the DnaJ-X subfamily; D represents the DUF3444 subfamily; E represents the Jiv90 subfamily; F represents the Fer4_8 subfamily.

We further analyzed the conserved motifs of ZmDnaJ proteins to evaluate their functional regions. Using the MEME website, 10 conserved motifs were identified, and their distribution in ZmDnaJ proteins, along with a phylogenetic tree is shown in Figure 2. We found that 32 ZmDnaJ proteins contain a complete J domain, including motifs 1, 2, 3, and 8. Among these, motif 1, with a stable HPD structure (the core of the J domain), is present in all proteins. Motifs 2 and 8, which form an α-helical structure, are located within the C-terminal region of the J-domain (Figure 2 and Figure S2). The remaining proteins only possess the HPD structure and an incomplete α-helical structure. In addition, some motifs are specific to particular groups, which might be related to distinct biological functions. For example, motifs 4, 5, and 6 are present in type I and type II, motif 7 is specific to the DnaJ-X subgroup, and motifs 9 and 10 are unique to the DUF3444 subgroup (Figure 2 and Figure S1). The above results would facilitate the prediction of *ZmDnaJ* gene functions.

Furthermore, the gene structure of the ZmDnaJ family was analyzed to better understand its characterization. The results showed significant variation in the number of exons (ranging from 1 to 22) and introns (ranging from 0 to 21) in individual *ZmDnaJ* genes (Figure 2). Most *ZmDnaJ* genes within the same group have similar exon numbers and structures. However, there are significant differences in intron lengths, as seen in *ZmDnaJ86* and *ZmDnaJ34* in type I; *ZmDnaJ24* and *ZmDnaJ60* in the DUF3444 subgroup, and *ZmDnaJ24* and *ZmDnaJ60* in another subgroup.

### 2.4. Gene Duplication Analysis of the ZmDnaJ Gene Family

**Figure 3:**
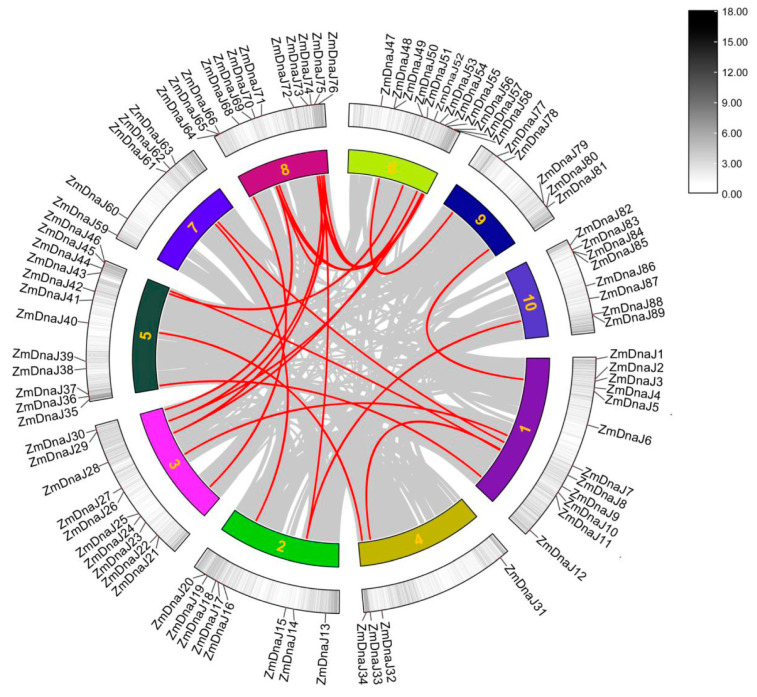
Gene duplication of *ZmDnaJ* genes on the 10 chromosomes of the maize genome. Red lines connect the duplicated gene pairs, while gray lines represent all synteny blocks within the maize genome. Detailed relationships of the duplicated genes are provided in Table S2.

To illustrate the replication patterns of *ZmDnaJ* gene family, we analyzed duplication events using MCScanX. We detected 18 pairs of duplication events involving 29 *ZmDnaJs* (Figure 3), indicating that these gene duplications contributed to the expansion of *the ZmDnaJ* gene family in maize. Further, we calculated nonsynonymous (Ka), synonymous substitutions (Ks), as well as the Ka/Ks ratios for these gene pairs to assess the evolutionary selection in the *ZmDnaJ* gene family. The results show that, except for three gene pairs (*ZmDnaJ14* and *ZmDnaJ75*; *ZmDnaJ14* and *ZmDnaJ88*; *ZmDnaJ48* and *ZmDnaJ77*), for which Ka/Ks values were not obtained, the Ka/Ks values of the remaining *ZmDnaJ* gene pairs are all less than 1, ranging from 0.091 to 0.615 (Table S2). This suggests that these genes have undergone purifying selection during evolution.

### 2.5. Cis-Regulating Elements in Maize ZmDnaJ Promoters

**Figure 4:**
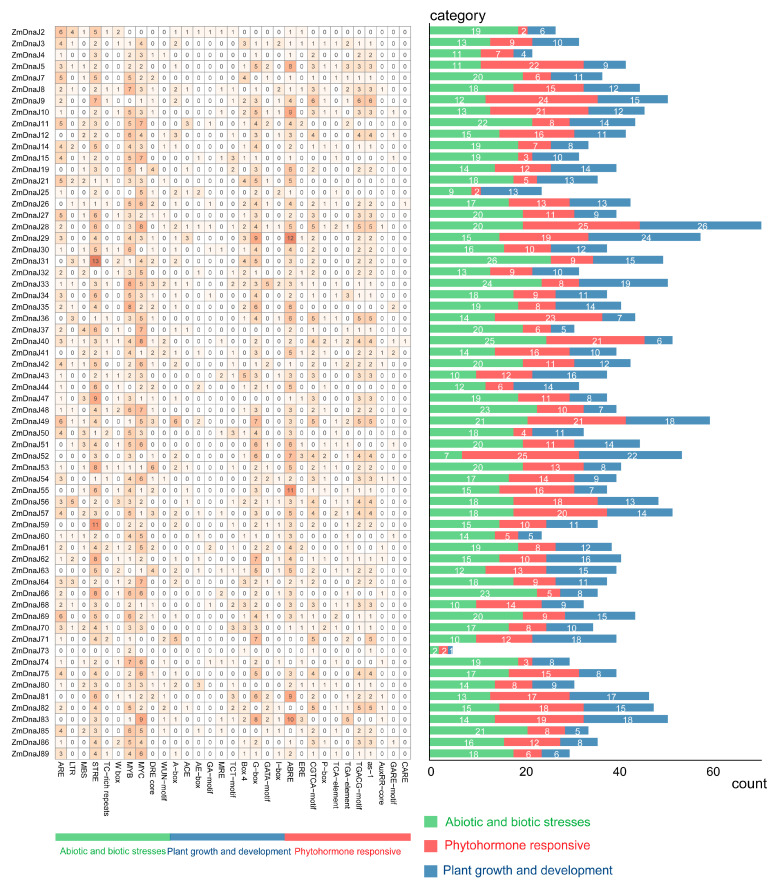
Cis-element number analysis in the *ZmDnaJ* gene family. The grid’s varying color intensities and numbers represent the number of different promoter elements within the *ZmDnaJ* genes. The histogram, with its different colors, shows the total number of cis-acting elements for each category.

The cis-regulatory elements of the proximal promoter play key roles in the initiation of gene expression. Therefore, we identified the conserved cis-elements present in the promoter of *ZmDnaJs* using PlantCARE [25]. A total of 66 conserved cis-elements were generated, as shown in Figure 4. These cis-elements could be classified into three categories: abiotic and biotic stress, plant growth and development, and phytohormone responsiveness. In the abiotic and biotic stress category, most elements are related to three general stress-responsive motifs: the stress-responsive element (STRE), MYB (CCAAT box), and MYC (CACATG box) binding sites, which account for 11%, 9.4% and 10% of the total identified cis-elements, respectively (Table S3). Other stress-responsive elements observed include the anaerobic-responsive element (ARE) motif, low-temperature-responsive (LTR) motifs, MYB binding site (MBS), TC-rich repeat, WRKY-box (W-box), dehydration-responsive element (DRE)-core, and the wound-responsive motif (WUN-motif). In the phytohormone-responsive category, 10 related cis-elements were identified, with the G-box occupying the highest percentage at 8.5%. In the phytohormone-responsive category, the ABRE element (~9.7%), involved in ABA responsiveness, was the most prominent, followed by two MeJA-responsiveness elements (the CGTCA motif (5.5%) and TGACG motif (5.5%)), as well as the as-1 motif involved in SA response (Figure 4, Table S3). These elements may play important roles in regulating the differential expression of *ZmDnaJs* during plant growth and development, especially under various biotic and abiotic stresses.

### 2.6. Expression Patterns of ZmDnaJ Gene Family Members in Different Tissues

**Figure 5:**
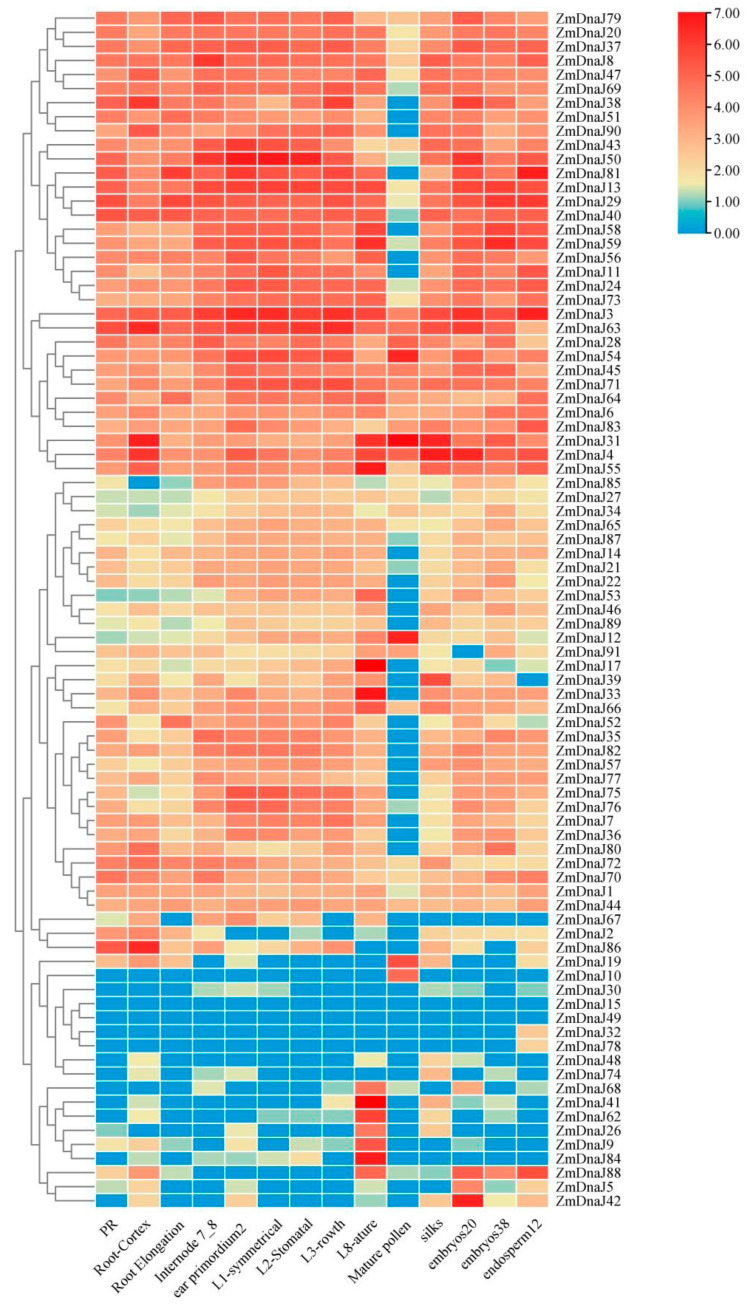
Expression pattern analysis of *ZmDnaJ* family genes in different tissues and developmental stages based on transcript data. The expression patterns of 91 genes were examined across 13 different tissues and developmental stages using public transcriptome data. The FPKM values (fragments per kilobase of transcript per million fragments) of all genes were transformed to the log2 scale. Red and blue colors indicate higher and lower relative transcript enrichment, respectively.

To assess the temporal expression patterns of *ZmDnaJs*, we investigated the transcriptome data of *ZmDnaJs* from various maize tissues and development stages, as reported by Walley et al. [26]. The expression level of each gene was normalized using the RPKM method. As shown in Figure 5, 39 *ZmDnaJs* were expressed across all tested tissues, including the root, internode, leaf, mature pollen, silk, embryos, and endosperm. Among these, 26 genes, such as *ZmDnaJ79*, *20*, *37*, *8*, *47*, *3*, and *63*, showed relatively high expression levels. Some *ZmDnaJs*, including *ZmDnaJ38*, *51*, *90*, *81*, *40*, *56*, *11*, and *14*, were not detected in mature pollen. The expression levels of *ZmDnaJ39*, *33*, *68*, *41*, *62*, *26*, *9*, *84*, and *88* were higher in the mature leaf than in the leaf zone (symmetrical), leaf zone 2 (stomatal), and leaf zone 2 (growth), indicating their involvement in leaf growth and development. In addition, pollen-specific expression was observed in *ZmDnaJ10*, while *ZmDnaJ32* and *ZmDnaJ78* were specifically expressed in the endosperms. *ZmDnaJ15* and *ZmDnaJ49* were barely detected in all tested tissues. The results indicated functional diversity among *ZmDnaJ* family members during maize growth and development.

### 2.7. Expression Patterns of ZmDnaJ Gene Family Members under Abiotic Stress

**Figure 6:**
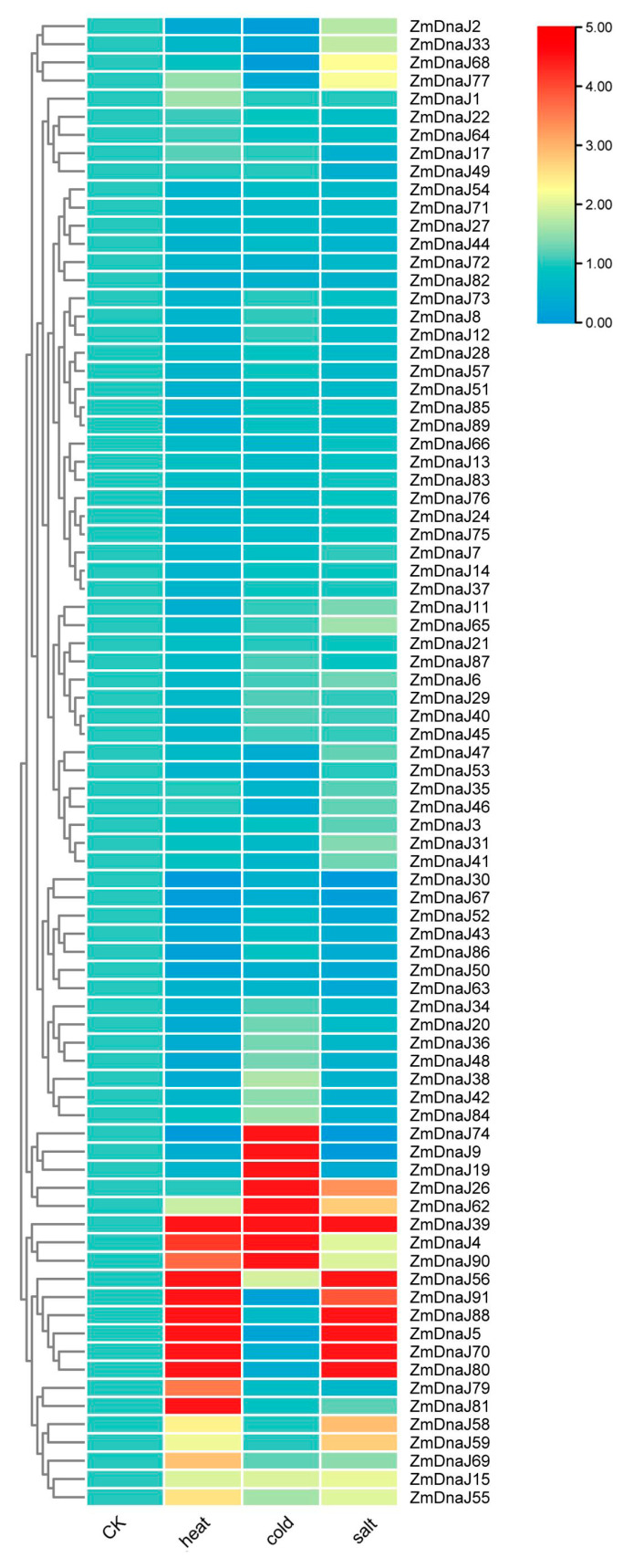
Gene expression profiles of *ZmDnaJ* genes in response to salt, heat, and cold stress treatments based on transcript data. The changes in expression of *ZmDnaJ* genes under salt, heat, and cold stress compared to normal conditions were calculated. Blue and red scales indicate low and high expression levels, respectively.

To analyze the expression patterns of *ZmDnaJs* in response to abiotic stress, we investigated the gene expression by using TBtools, based on transcriptome data reported by Makarevitch et al. [27]. Relative expression values were calculated by comparing treated samples to control samples. The results showed that, compared with the control group, 12 genes (*ZmDnaJ39*, *62*, *4*, *90*, *56*, *91*, *88*, *5*, *70*, *80*, *58*, and *59*) were upregulated, while 15 genes (*30*, *67*, *52*, *43*, *86*, *50*, *63*, *48*, *38*, *42*, *19*, *9* and *74*) were downregulated under heat and salt stresses. The parallel expression profiles of *ZmDnaJs* under these conditions suggested an extensive overlap between heat and salt stress response pathways. In response to cold stress, eight genes, (*ZmDnaJ19*, *9*, *74*, *26*, *62*, *39*, *4*, and *90*) showed more than a two-fold upregulation, while five genes, (*ZmDnaJ88*, *5*, *2*, *33*, and *68*) exhibited more than a two-fold downregulation. Notably, *ZmDnaJ39* was upregulated in response to salt, heat, and cold stresses (Figure 6). These findings indicate that *ZmDnaJ* genes play complex and significant regulatory roles in response to various abiotic stresses in maize, with extensive overlap between the pathways responding to heat and salt stresses.

### 2.8. Expression Patterns of 17 ZmDnaJs of Five Inbred Maize Lines under Heat Stress

**Figure 7:**
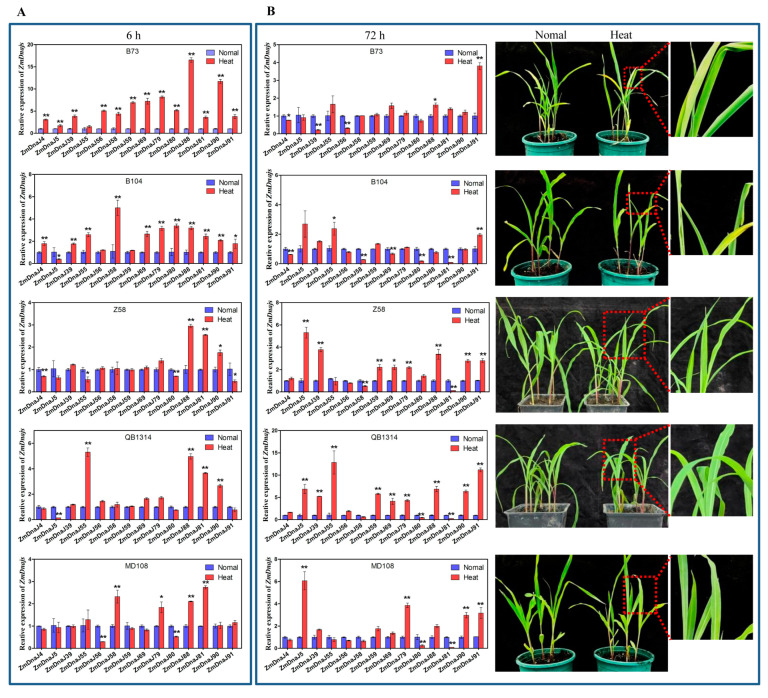
Relative expression levels of 14 selected *ZmDnaJ* genes and the phenotypes of five inbred maize lines under heat treatment. (**A**) The relative expression levels of 14 *ZmDnaJ* genes in the seedling leaves of five inbred lines were measured using qRT-PCR, with ZmUbi as the reference gene. Results are shown as mean ± standard deviation, with significance indicated by * *p* < 0.05 and ** *p* < 0.01 (Student’s *t*-test). Leaf samples were collected 6 h after heat treatment. (**B**) Left panel: Relative expression levels of the 14 *ZmDnaJ* genes in seedling leaves at 72 h after heat treatment. Right panel: Phenotypes of the five inbred lines (B73, B104, Zheng 58, QB1314, and MD108) at 72 h after heat treatment.

To further explore the potential roles of the 16 upregulated *ZmDnaJs* (*39*, *4*, *90*, *56*, *91*, *88*, *5*, *70*, *80*, *79*, *81*, *58*, *59*, *69*, *15*, and *55*) in heat stress tolerance in maize, we conducted qRT-PCR to assess their expressions before and after heat treatment at the seedling stage across five inbred lines. RNA quality (Figure S3, Table S4) and melting curves (Figure S4) ensured the reliability of the qRT-PCR results. The results showed that, compared to normal conditions, nearly all *ZmDnaJs* showed significantly increased expressions in B73 and B104 at 6 h after heat treatment, which was consistent with the transcriptome data. In the other inbred lines, only a few *ZmDnaJs* were upregulated at this time point. For example, *ZmDnaJ88*, *81*, and *90* were upregulated in Z58, *ZmDnaJ55*, *88*, *81*, and *90* in QB1314, and *ZmDnaJ58*, *79*, *88*, and *81* in MD108, with two- to five-fold increases (Figure 7A). Additionally, *ZmDnaJ70*, and *15* did not show detectable expression.

After 72 h of continuous heat stress, the leaves of B73 and B104 were severely curled or wilted, whereas Z58, QB1314, and MD108 still grew normally (Figure 7B). Notably, qRT-PCR results showed that, except for *ZmDnaJ91*, the expression levels of most *ZmDnaJs* in B73 and B104 returned to normal levels after 72 h of heat treatment, with some even lower than untreated levels. However, in Z58, QB1314, and MD108, many *ZmDnaJs*, including *ZmDnaJ55*, *79*, *88*, *90*, and *91*, were upregulated after 72 h of heat treatment, with expression levels increasing by 2- to 12-fold compared to normal conditions (Figure 7B, Table 1), indicating these *ZmDnaJs* function against prolonged heat stress. The above results suggest that multiple *ZmDnaJ* genes may enhance maize heat tolerance through synergistic interactions.

## 3. Discussion

DnaJ proteins, as types of heat shock proteins, are widely present in plant tissues and cells as stress response factors. They play important roles in plant responses to both biotic and abiotic stresses. For instance, the overexpression of a tobacco J-domain protein was shown to enhance drought tolerance in transgenic *Arabidopsis* [28]. The tomato chloroplast-targeted DnaJ protein was found to protect Rubisco activity under heat stress [29]. The identification and characterization of the *DnaJ* gene family have been reported in many plant species, such as *Arabidopsis*, rice, cucumber, pepper, *sorghum bicolor*, and *Catalpa bungei* [8,9,23,24,30,31]. In this study, we identified 91 *ZmDnaJ* genes in maize using bioinformatics methods and conducted a comprehensive analysis of their phylogeny, sequence structure, conserved domain, collinearity, and expression patterns during different tissues and under abiotic stresses. This research provides a theoretical foundation and valuable data for further functional studies.

Previous studies have indicated that ZmDnaJ proteins facilitate the refolding of proteins damaged by stress and regulate the activity of many regulatory proteins in various cellular compartments, including the mitochondrial matrix, the lumen of the endoplasmic reticulum, the cytosol, and the nucleus [8,9]. For example, it is reported that chloroplast-localized DnaJ proteins are well-characterized as key mediators in plant stress responses [23,24,32]. Bekh-Ochir et al. reported that the mitochondrial DnaJ/Hsp40 family protein BIL2 promotes plant growth and resistance against environmental stress in brassinosteroid signaling [33]. Our subcellular localization predictions for ZmDnaJ proteins (Table S1) similarly indicated their probable role in maize’s response to environmental stress.

The phylogenetic relationship of gene family members helps elucidate their evolutionary history, classification, functional prediction, and the adaptation and speciation processes in both organisms and genes. J-proteins have traditionally been classified into three types (type I, type II, and type III) based on the bacterial DnaJ classification [34]. The signature of all DnaJs is the J-domain, characterized by a highly conserved and functionally essential HPD motif. Type I and Type II proteins also contain a conserved C-terminal region, with Type I additionally characterized by zinc finger domains. Type III proteins can be further classified based on secondary structural elements, such as tetratricopeptide repeats and Fe-S clusters [8,9]. Further studies have identified “J-like proteins” that have significant sequence and structural similarities to the J-domain but lack the HPD motif [6]. In our study, all 91 identified ZmDnaJ proteins contained the HPD motif, with no J-like proteins detected (Figure 2 and Figure S2), likely due to the exclusion of sequences that did not have a fully conserved domain. We classified the 91 *ZmDnaJs* into three types: eight in Type I, twelve in Type II, and the remaining in Type III, consistent with the clustering observed in *Arabidopsis* and other plants [9,13]. Gene duplication is recognized as a major evolutionary force in the development of genetic systems and genomes [35]. In evolutionary biology, the ratio of Ka/Ks in a gene can provide insights into the type of selection acting on that gene. A Ka/Ks ratio of more than one indicates positive selection, a ratio of one implies neutral evolution, and a ratio of less than one suggests purifying selection [36]. Our results showed that 18 pairs of *ZmDnaJ* genes, located on different chromosomes, have undergone successive duplication events (Figure 3), indicating gene expansion during evolution. The Ka/Ks ratio for all duplicated genes was less than one (Figure 3), suggesting that these gene pairs may have undergone purifying selection, resulting in limited functional divergence following duplication.

The cis-elements within promoters play a crucial role in initiating gene expression. Our analysis of the cis-acting elements within promoters of *ZmDnaJ* genes revealed a diverse array of regulatory elements involved in plant growth, stress responses, and phytohormone signaling (Figure 4). Elements such as STRE, MYB, MYC, and ABRE in the promoters of *ZmDnaJ* genes have been shown to be involved in complex regulatory networks that govern stress tolerance [37,38,39]. Additionally, RNA-seq data [26,27] analysis revealed distinct expression patterns of *ZmDnaJ* genes across different tissues, developmental stages, and in response to three stresses (Figure 5 and Figure 6). This is consistent with the established roles of cis-elements in modulating gene expression in response to various internal and external cues. The distinct tissue-specific and stress-responsive expression patterns observed in RNA-seq data suggest that *ZmDnaJ* genes are finely regulated to respond to different physiological conditions, indicating their functional diversification and critical roles in maize adaptability.

High temperature has emerged as one of the most critical abiotic stresses impacting crop plants. Heat shock proteins (Hsps), including Hsp100, Hsp90, Hsp70, Hsp60, Hsp40, and Hsp20, are key components of the heat shock regulatory network that help plants cope with this stress [40]. Our study revealed the differences in expression patterns of *ZmDnaJ* genes and their response to heat stress among five inbred lines. The heat-tolerant inbred lines (QB1314, MD108, and Zheng58) show a greater number of upregulated *ZmDnaJ* genes and higher expression levels compared to the heat-sensitive lines (B73 and B104) after 72 h of heat stress (Figure 7, Table 1). These indicated that they may function synergistically to provide sustained protection against prolonged heat stress. Apart from heat stress, *Hsp* expression was strongly induced by cold, salt, and drought stress [41]. Moreover, many *Hsps* had highly similar or overlapped response and regulation patterns under different stresses [41,42,43]. Here, we found that many *ZmDnaJs* exhibited high similarities in expression patterns in response to heat and salt stresses (Figure 6). These suggested considerable cross-talk among the regulatory networks of various stresses.

In conclusion, the differential expression of *ZmDnaJ* genes and their responses to various stresses indicate their critical roles in maize adaptation. The above analysis offers a theoretical basis for future functional validation of these genes.

## 4. Materials and Methods

### 4.1. Genome-Wide Identification of ZmDnaJ Gene Family in Maize

The genome sequences of maize were downloaded from the MaizeGDB database (https://www.maizegdb.org/ (accessed on 23 October 2023)). Subsequently, the hidden Markov model (HMM) profile of the J-domain (PF00226) was downloaded from the Pfam database (http://pfam.xfam.org/ (accessed on 23 October 2023)) to search the putative protein sequence containing DnaJ domain in maize (e-value < 10^−10^). To validate the accuracy of these DnaJ proteins, the redundant protein sequences were removed manually, and then the remaining protein sequences were confirmed as the final ZmDnaJ protein sequences based on the Conserved Domain Database (CDD) of NCBI (https://www.ncbi.nlm.nih.gov/cdd/ (accessed on 23 October 2023)). Molecular weight, theoretical isoelectric point (pI), Instability Index, and Grand Average of Hydropathicity of the *ZmDnaJ* gene family members were analyzed with EXPASY PROTOPARAM (https://web.expasy.org/protparam/ (accessed on 2 November 2023)). The WoLF PSORT (https://wolfpsort.hgc.jp/ (accessed on 8 November 2023)) was used to predict protein subcellular localization.

### 4.2. Phylogenetic Tree and Conserved Domain Analysis of ZmDnaJ Gene Family

To explore the evolutionary relationship of the *ZmDnaJ* gene family between maize and *Arabidopsis*, the DnaJ protein sequences from *Arabidopsis* and 89 *ZmDnaJs* were used for the phylogenetic tree construction with MEGA-X (v1.2.6) software [44]. The phylogenetic tree was constructed using the neighbor-joining phylogenetic tree (NJ) and the Jones–Taylor–Thorton (JTT) model with 1000 bootstraps. The phylogenetic tree was modified using the online software iTOL (https://itol.embl.de/ (accessed on 8 November 2023)) [45]. To understand the relationship between evolution and conserved domains among ZmDnaJ proteins, the conserved domains of ZmDnaJ proteins were obtained using the NCBI-CDD website. The above files were optimized using TBtools (v2.096) [46].

### 4.3. Analysis of Protein Motif, Gene Structure, and Gene Duplication of the ZmDnaJ Gene Family

The gff3 file (Zea mays. Zm-B73-REFERENCE-NAM-5.0.51.gff3), downloaded from NCBI, was used to analyze the gene structure of *ZmDnaJ* genes. The motifs of ZmDnaJ proteins were analyzed via the online MEME tool (http://meme-suite.org/tools/meme (accessed on 15 November 2023)) based on their protein sequences (the number of different motifs, 10; minimum width of motifs, 6; maximum width of motifs, 50). To identify the gene duplication pattern, ZmDnaJ genes in maize were analyzed using the one-step MCScanX tool from TBtools with default parameters, including an E-value cut-off < 1 × 10^−10^ and the number of BLAST hits at 5.

### 4.4. Cis-Acting Element Analysis of ZmDnaJ Genes

The upstream regulatory regions (~2000 bp) of *ZmDnaJ* gene sequences were retrieved from the MaizeGDB database and subsequently used to search for cis-regulating elements in the PlantCARE website https://bioinformatics.psb.ugent.be/webtools/plantcare/html/ (accessed on 3 December 2023) [25].

### 4.5. Expression of ZmDnaJ Genes Based on RNA-Seq in Maize cv. B73

In this paper, RNA-seq data reported by Walley et al. [26] were used to investigate the expression patterns of putative *ZmDnaJ* genes in maize. Different tissues were selected: primary root 5 days, root cortex 5 days, root elongation zone 5 days, 7–8 internode, ear primordium 2–4 mm, leaf zone 1 (symmetrical), leaf zone 2 (Stomatal), leaf zone 3 (growth), mature leaf 8, mature pollen, silk, embryo 20 days after pollination (DAP), embryo 38 DAP, endosperm crown 12 DAP. RPKM values (reads per kilo bases per million mapped reads) of *ZmDnaJ* genes were log2-transformed. Additionally, we also analyzed the change of expression of *ZmDnaJ* genes under salt, heat, and cold stress based on RNA-seq data reported by Makarevitch et al. [27]. Heat maps of *ZmDnaJ* genes in different tissues or under abiotic stresses were performed using the software TBtools (v2.096) [46].

### 4.6. Plant Material and Stress Treatment

The maize inbred lines, including B73, B104, QB1314 (obtained from Guizhou Academy of Agricultural Sciences, Guiyang, China), MD108, and Zheng58, were grown in a chamber (JIUPO, Fuzhou, China; Catalog: BPC500H) under normal conditions (32 °C/22 °C, day/night) with a photoperiod of 14 h light and 10 h dark. The light intensities were ramped up to 300 μmol/m^2^/s. Half of the plants at the two-leaf stage (V2, 10 days after seed germination) were cultivated under consistent conditions to ensure their growth and development, while others were moved to a higher temperature circumstance (42 °C/32 °C, day/night) for the heat stress treatment. The photoperiod and light intensities under the higher temperature conditions were set as the control conditions. In order to avoid drought stress, the trays were adequately watered during the treatment. Samples were collected from the plants under the two conditions after 6 or 72 h of treatment for the mRNA-seq. Leaves under each condition were collected and stored at −80 °C after the evaporation of liquid nitrogen.

### 4.7. RNA Extraction, and Quantitative Real-Time PCR Analysis (qRT-PCR)

Total RNA was extracted using RNA-easy Isolation Reagent (Vazyme, Nanjing, China) and reverse-transcribed using HiScript III RT SuperMix kit qPCR (+gDNA wiper) (Vazyme, Nanjing, China), the operational procedure followed the manufacturer’s procedure. qRT-PCR was performed on ABI Prism 7500 Sequence Detection System (Applied Biosystems) using 2× Taq Master Mix (Vazyme, Nanjing, China) in 10 μL qRT-PCR reactions. The UBI gene was used as an internal control [47]. The relative expression values of genes were calculated using the 2^−ΔΔCt^ method [48]. Three biological repeats were performed for each sample. The primers for qRT-PCR are listed in Table S5.

## 5. Conclusions

This study identified 91 *ZmDnaJ* genes in the maize genome and analyzed their physicochemical properties, phylogenetic relationships, conserved motifs, gene structures, and expression patterns. The *ZmDnaJ* genes showed significant diversity in their expression across different tissues, developmental stages, and in response to various abiotic stresses, including heat, salt, and cold. Our findings highlight the potential role of *ZmDnaJ* genes in enhancing stress tolerance, with certain genes, such as *ZmDnaJ39*, consistently upregulated under multiple stresses. Additionally, heat-tolerant maize inbred lines exhibited higher expression levels of multiple *ZmDnaJ* genes compared to heat-sensitive lines, suggesting a synergistic function in stress response. These insights provide a valuable foundation for further exploration of *ZmDnaJ* genes in improving maize resilience to environmental stresses.

## Figures and Tables

**Table 1:** The expression profiles of 14 selected *ZmDnaJ* genes of five maize inbred lines under heat treatment.

Heat Stress Gene	Heat-Sensitive Inbred Lines				Heat-Tolerant Inbred Lines					
	B73		B104		Z58		QB1314		MD108	
	6 h	72 h	6 h	72 h	6 h	72 h	6 h	72 h	6 h	72 h
*ZmDnaJ4*	up	-	up	down	down	-	-	-	-	-
*ZmDnaJ5*	up	-	down	-	-	up	down	up	-	down
*ZmDnaJ39*	up	down	up	-	-	up	-	up	-	-
*ZmDnaJ55*	up	-	up	up	down		up	up	-	-
*ZmDnaJ56*	up	down	-	-	-	-	-		down	-
*ZmDnaJ58*	up	-	up	down	-	down	-		up	-
*ZmDnaJ59*	up	-	-	-	-	up	-	up	-	up
*ZmDnaJ69*	up	-	up	-	-	up	-	up	-	-
*ZmDnaJ79*	up	-	up	-	-	up	-	up	up	up
*ZmDnaJ80*	up	-	up	down	down	-	-	-	down	-
*ZmDnaJ81*	up	-	up	down	up	down	up	down	up	-
*ZmDnaJ88*	up	up	up	-	up	up	up	up	up	up
*ZmDnaJ90*	up	-	up	-	up	up	up	up	-	up
*ZmDnaJ91*	up	up	up	up	down	up	-	up	-	up

Note: red box represents up-regulated, green box represents down-regulated, - indicates no difference.

## Data Availability

Data are contained within the article and Supplementary Materials.

## Supplementary Materials

The following supporting information can be downloaded at https://www.mdpi.com/article/10.3390/plants13172488/s1, Figure S1 Phylogenetic relationship and conserved domain of ZmDnaJ members. Different colored boxes represent different subfamilies, while black lines indicate non-conserved sequences. Figure S2 Sequence logos of J-domains of ZmDnaJ proteins. The J-domains include motifs 1, 2, 3, and 8. Asterisks indicate the conserved histidine/proline/aspartic (HPD) residues in the J-domains among ZmDnaJ proteins. Figure S3 The electropherograms of RNA bands in agarose gels from the maize inbred lines. Figure S4 Melting curves of transcripts targeted for qRT-PCR. Table S1. The list of *ZmDnaJ* members identified in maize. Table S2. Inference of duplication time in paralogous pairs. Table S3. Statistics of the Proportions of four cis-Elements distributed in the promoters of ZmDnaJ genes. Table S4. The information regarding quality of total RNA samples. Table S5. Primers used in this study.
