# Supplementary figures and images for: Genome-Wide Identification and Analysis of Maize DnaJ Family Genes in Response to Salt, Heat, and Cold at the Seedling Stage
Source: Plants (Basel). 2024 Sep 5;13(17):2488. doi: 10.3390/plants13172488 (PMC11396969; doi:10.3390/plants13172488)

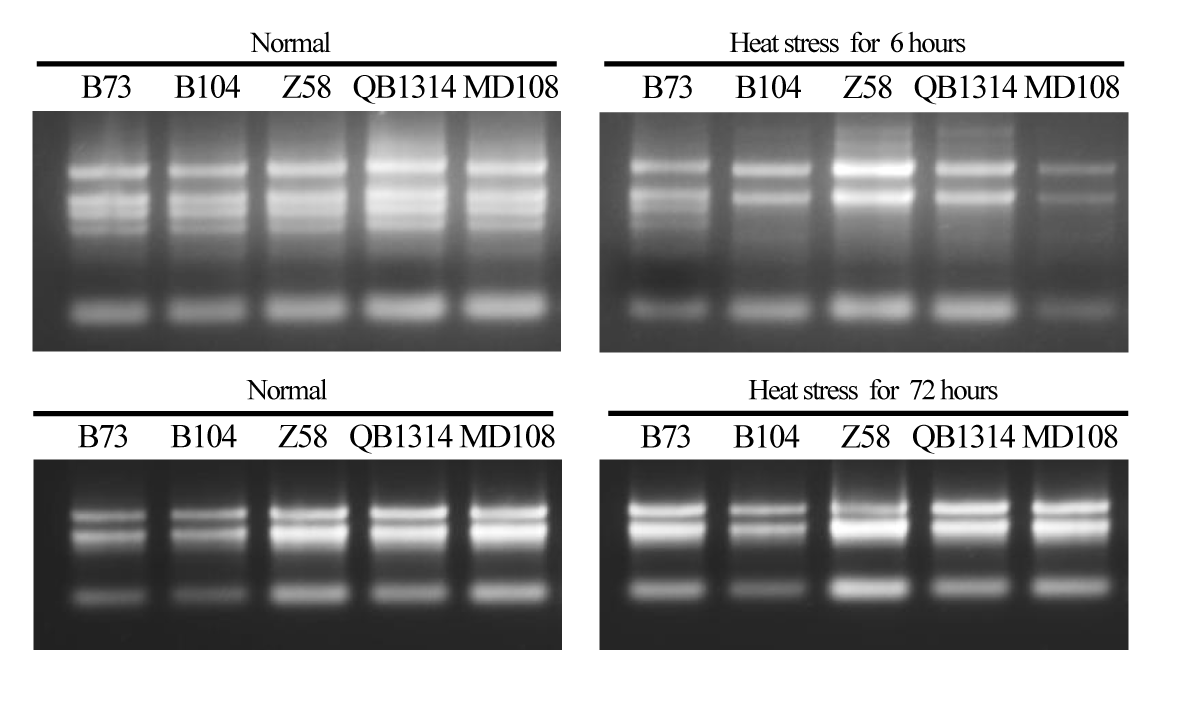

Supplement: Supplementary file 1 [file plants-13-02488-s001.zip › Figure S3.tif]

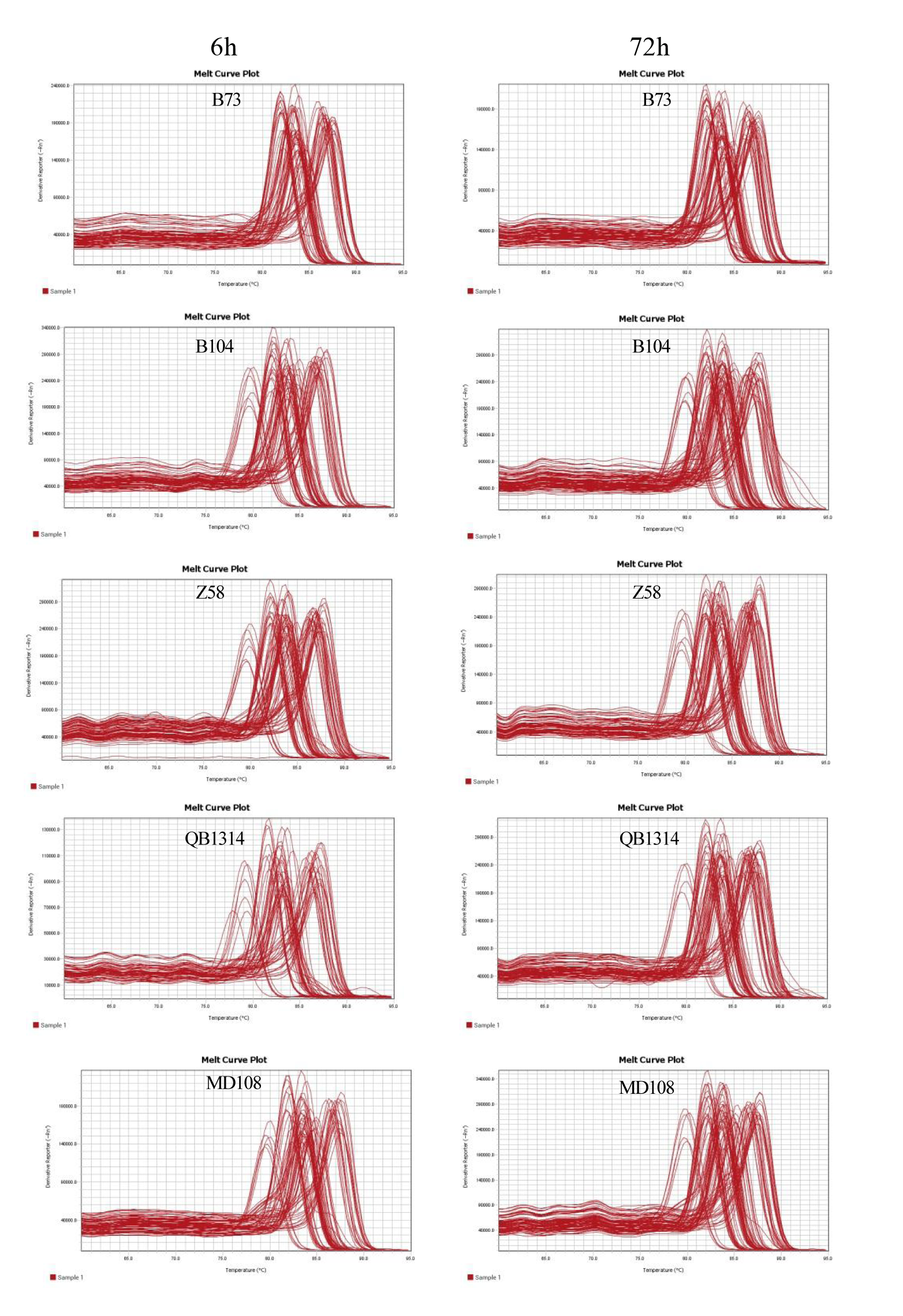

Supplement: Supplementary file 1 [file plants-13-02488-s001.zip › Figure S4.tif]

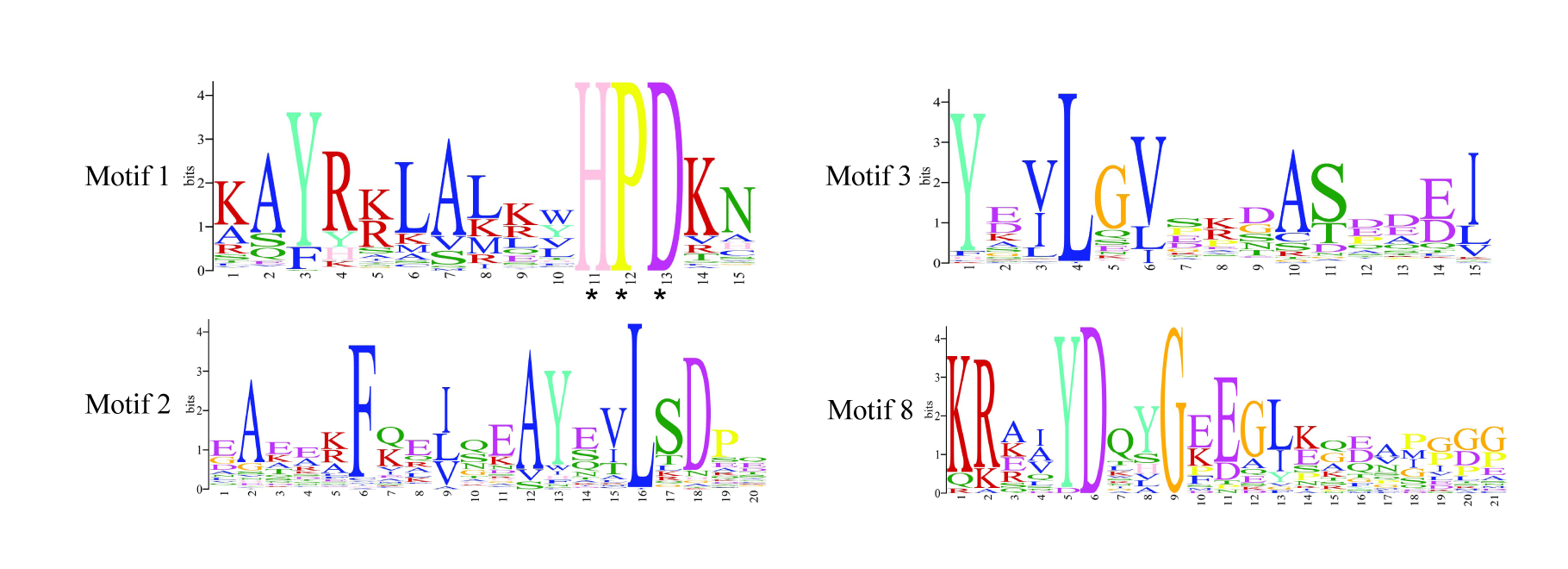

Supplement: Supplementary file 1 [file plants-13-02488-s001.zip › Figure S2.tif]

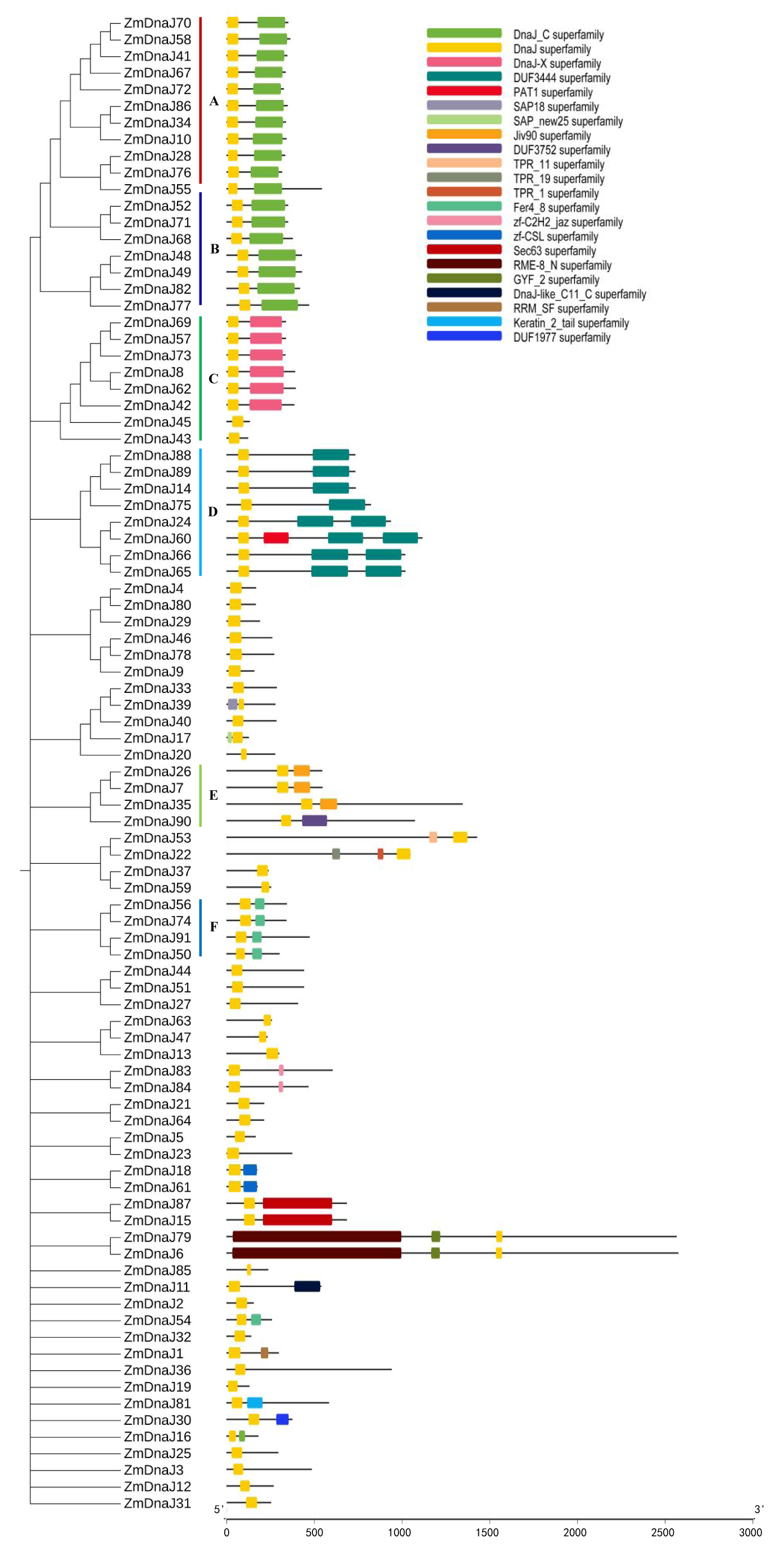

Supplement: Supplementary file 1 [file plants-13-02488-s001.zip › Figure S1.tif]
